# Associations between e-Health literacy, self-efficacy, and quality of life among hemodialysis patients: A cross-sectional study from Northern Jordan

**DOI:** 10.1371/journal.pone.0354170

**Published:** 2026-08-04

**Authors:** Rawand A. Khasawneh, Khalid Kheirallah, Rawan Ahmad Al-Laheem, Samah Al-Shatnawi, Mohammad Nusair

**Affiliations:** 1 Department of Clinical Pharmacy, Jordan University of Science and Technology, Irbid, Jordan; 2 Department of Public Health, Jordan University of Science and Technology, Irbid, Jordan; 3 Pharmacy Practice Department, Faculty of Pharmacy, Yarmouk University, Irbid, Jordan; Japanese Red Cross Medical Center, JAPAN

## Abstract

**Introduction:**

Chronic kidney disease (CKD) is a growing global health issue that significantly impairs quality of life (QoL), particularly among patients undergoing dialysis. This study examines the relationships between e-health literacy, self-efficacy, and QoL in this vulnerable population.

**Objectives:**

To examine associations among e-health literacy, self-efficacy, and health-related quality of life (HRQoL) among patients receiving hemodialysis in Northern Jordan.

**Methods:**

A cross-sectional study was conducted among 184 adult HD patients recruited from four hospitals in Northern Jordan. Data were collected using validated Arabic versions of the eHealth Literacy Scale (eHEALS), the Self-Efficacy for Managing Chronic Disease Scale (SEMCD-6), and the Kidney Disease Quality of Life-36 (KDQOL-36). Spearman correlation was used to examine associations between variables. Multiple linear regression was performed to identify independent predictors of KDQOL-36 total scores.

**Results:**

Among 184 participants (mean age 49.9 years, 65% male), the mean self-efficacy score was 25.52 (SD = 1.8) and the mean e-health literacy score was 3.62 (SD = 0.58). The mean KDQOL‑36 total score was 105 ± 17, with domain scores of 39 ± 7 for Symptoms/Problems, 25 ± 6 for Effects of Kidney Disease, 11 ± 3 for Burden of Kidney Disease, 29 ± 5 for Social Function, 12 ± 3 for the Physical Component Summary, and 21 ± 4 for the Mental Component Summary. Multivariable regression identified independent predictors of higher quality of life: graduate-level education (B = 8.06, 95% CI: 2.32–13.80, p = 0.006) and employment (B = 7.14, 95% CI: 2.10–12.20, p = 0.006). Diabetes was independently associated with lower quality of life (B = −5.92, 95% CI: −10.90 to −0.95, p = 0.020). Although significant in unadjusted analyses, e-health literacy and self-efficacy were not independent predictors after adjustment for socioeconomic and clinical factors.

**Conclusion:**

Health-related quality of life among HD patients appears to be influenced primarily by socioeconomic and clinical factors, particularly educational attainment, employment status, and diabetes mellitus. Although e-health literacy and self-efficacy were associated with HRQoL in unadjusted analyses, these associations were attenuated after adjusting for socioeconomic and clinical characteristics. Interventions that address educational disparities, support patient engagement, and optimize comorbidity management may improve health outcomes among patients receiving hemodialysis.

## Introduction

End-stage renal disease (ESRD) affects 6,708 patients in Jordan, with 97.6% receiving hemodialysis (HD) [1]. Patients face diminished quality of life (QoL) due to treatment complexity, comorbidities, and limited self-management skills [2,3]. e-Health literacy (eHL)—defined as the ability to seek, understand, and apply electronic health information [4]—and self-efficacy (confidence in managing health tasks) [5], are critical for chronic disease management. While global studies link these factors to improved outcomes [6], data in Arab HD populations remain scarce. This study examines how eHL and self-efficacy interact to influence QoL in Jordanian HD patients.

## Methodology

### Study design and participants

A cross-sectional study (April 16–September 7, 2023) enrolled 184 adult HD patients from four Northern Jordan hospitals: King Abdullah University Hospital, Princess Bassma Hospital, Al Mafraq Government Hospital, and Northern Badia Hospital. Inclusion criteria: (1) age 18–85 years, (2) Arabic literacy, (3) ≥3 months on HD. Exclusion criteria: cognitive impairment or severe psychiatric conditions.

### Study instruments

Several validated tools were employed to assess the variables in this study, including the eHL Scale (eHEALS), the KDQOL-36, and the Self-Efficacy of Chronic Disease Scale.

1. **General Sociodemographic Questionnaire**A sociodemographic questionnaire was used to collect data on variables such as age, sex, marital status, education level, occupation, income, and living area (urban or rural). Information on occupation status, duration since kidney disease diagnosis, dialysis frequency, and device use for health-related internet access was also collected.
2. **E-Health Literacy Scale (eHEALS)**The eHEALS assesses individuals’ ability to seek, find, understand, and evaluate health information from the internet. The Arabic version of eHEALS has been validated for use in Arabic-speaking populations [7]. This scale includes 8 items, with responses scored on a 5-point Likert scale, ranging from 1 (strongly disagree) to 5 (strongly agree). Higher scores indicate greater eHL.
3. **Kidney Disease Quality of Life (KDQOL-36)**The KDQOL-36 is a disease-specific quality of life tool for patients with chronic kidney disease, including hemodialysis patients. The Arabic version of this tool has been validated and used among Arab populations [8]. It contains the following domains:
  - **Physical Composite Scale (PCS):** This scale assesses patients’ overall physical health and includes questions regarding physical functioning, pain, and limitations in daily activities. Higher scores indicate better physical health.
  - **Mental Composite Scale (MCS):** This scale measures psychological well-being, including mood, emotional distress, and mental functioning. Higher scores indicate better mental health.
  - **Burden of Kidney Disease (BKD):** This tool evaluates the patient’s perception of how kidney disease affects their daily life and emotional state. Higher scores suggest a lower perceived burden.
  - **Symptom/Problem List (SP):** Assesses the severity and frequency of symptoms experienced by the patient, including fatigue, pain, and other kidney disease-related symptoms. A lower score indicates fewer symptoms.
  - Effects of Kidney Disease (KDE): Measures how kidney disease affects patients’ quality of life, specifically on their physical and social functioning. Higher scores indicate a lower perceived impact of kidney disease on life quality.

The KDQOL-36 tool is scored using standardized algorithms for each domain, with higher scores indicating a better health-related quality of life. The total score is calculated from the responses, with higher scores reflecting improved health-related quality of life.

1. Self-Efficacy of Chronic Disease ScaleThe Self-Efficacy of Chronic Disease Scale was used to assess patients’ self-efficacy in handling their chronic kidney disease [9]. The scale consists of six questions, each evaluating the patient’s confidence in managing various aspects of their disease [9]. The validation was conducted by **Khraim, Munir, Johnson, Alqudah, and Kan’an (2022)**, who translated, culturally adapted, and psychometrically tested the SEMCD among Arabic‑speaking patients with multimorbidity, including chronic kidney disease. Their study confirmed the scale’s reliability and validity, demonstrating excellent internal consistency (Cronbach’s α = 0.949) [10]. The scale is scored on a 10-point Likert scale, with higher scores indicating greater confidence in managing the disease. This scale is widely used to evaluate the self-efficacy of patients dealing with chronic health conditions, including CKD.

To ensure the relevance, clarity, and cultural appropriateness of the study instruments, a face and content validation process was conducted prior to the main data collection. The tools used in this study, including the eHEALS**,** KDQOL-36, and Self-Efficacy Scale, were reviewed for comprehensibility and suitability for the target population (hemodialysis patients in Jordan). The face validation involved a preliminary review of the questionnaires by a small group of 10 hemodialysis patients, who were asked to provide feedback on the clarity of the questions, the appropriateness of the language, and the overall ease of understanding. This group of patients was selected to represent a range of educational backgrounds and experiences, ensuring the questionnaires were understandable and relevant to the diverse patient population.

The tools were reviewed by a panel of five experts from various fields for content validation. This panel consisted of a psychiatric specialist, two pharmacists**,** a nurse, and a public health professor. These experts evaluated the tools based on their relevance to the study’s objectives, the appropriateness of the items for measuring the desired constructs (e.g., eHL, quality of life, and self-efficacy), and the cultural sensitivity of the questions. The experts’ feedback led to minor wording revisions to enhance clarity and ensure that target population would easily understand the instruments. After these adjustments, the tools were finalized for use in the study. The validation helped confirm that the instruments were both convenient and culturally appropriate for the study population, ensuring that the data collected would be valid and reliable.

Moreover, Cronbach’s alpha for each scale was calculated to ensure the reliability and internal consistency of the tools used in this study. The self-efficacy scale demonstrated good internal consistency with a Cronbach’s alpha of 0.83. The eHEALS questionnaire showed excellent internal consistency, with a Cronbach’s alpha of 0.94. The KDQOL-36 scale also displayed acceptable internal consistency, with a Cronbach’s alpha of 0.79. According to previous research, a Cronbach’s alpha of 0.7 or higher is considered acceptable [11], supporting the reliability of the scales used in this study. These values confirm that the tools used in the study are reliable and consistent in measuring the intended constructs.

### Statistical analysis

Data were analyzed using SPSS v22.0. Descriptive statistics expressed as mean ± SD or frequencies. Spearman’s correlation assessed relationships between variables. Linear regression identified QoL predictors (p < 0.05 significance).

### Ethical approval

The study was approved by the Institutional Review Board at King Abdullah University Hospital (approval number: 2023/159/14). Written informed consent was obtained from study participants.

## Results

### Demographic characteristics

A total of 184 dialysis patients participated in the study, with a mean age of 49.9 ± 14.3 years. Approximately 65% of the participants were male (n = 119), and 73.8% were married. More than half of the participants lived in rural areas (55.2%, n = 101). The participants’ educational backgrounds varied: 42.1% (n = 77) had completed primary education, 27.9% (n = 51) had completed secondary school, and 30.1% (n = 55) earned a graduate degree. Most respondents (96.2%, n = 177) worked in non-medical fields. Regarding physical activity, 95.1% of participants (n = 175) exercised three times per week.

Regarding disease-specific characteristics, the mean duration of dialysis treatment was 3.48 years, and most participants received dialysis three times a week. HTN was the most common comorbidity (82.15%), followed by DM (45.1%) and cardiovascular disease (12%) (Table 1). Regarding internet use, most participants had home internet access (80%) and moderate to high internet skills.

**Table 1:** Socio-demographic characteristics of CKD patients (N = 184).

Characteristic	Mean±SD	N(%)
Age	49.9 ± 14.3	
**Sex** Male Female		119(65%) 64(35%)
**Marital status** Single Married Other		36(19.7%) 135(73.8%) 12(6.5%)
**Education level** Primary education / no formal education Secondary school Post-Secondary		77(42.1%) 51(27.9%) 55(30.1%)
**Occupation filed** Medical Non-medical		7(3.8%) 117(96.2%)
**Income** Less than 300 JOD 300-500 JOD 500-1000 JOD Above 1000 JOD		53(28.8%) 59(32.1%) 67(36.4%) 5(2.7%)
**Living** Rural Urban		101(55.2%) 82(44.8%)
**Occupation** Employed-Full time Employed- Part time Retired No work		45(24.5%) 13(7.1%) 43(23.4%) 83(45.1%)
**Frequency of dialysis/week** Twice Three time Four		8(4.3%) 175(95.1%) 1(0.5%)
**Duration on dialysis (years)**	3.48 ± 0.52	
**Comorbidities disease** HTN DM CVD Other		151(82.15) 83(45.1%) 22(12%) 9(4.9%)

### Self-efficacy

Self-efficacy scores ranged from 1 to 10, with a mean score of 25.52 (range 6–60). Participants reported varying levels of confidence in managing different aspects of their dialysis treatment. The mean score for confidence in preventing disease-related fatigue from interfering with activities was 5.71 ± 1.7, and the mean score for confidence in managing physical discomfort or pain was 5.39 ± 1.8. Confidence in managing emotional distress had a mean score of 5.36 ± 1.64, and the mean overall confidence in managing health tasks was 5.43 ± 1.8. Confidence in using non-medical methods to limit the disease’s impact was 5.81 ± 1.7. These moderate scores suggest that participants felt somewhat confident in managing their disease across different areas (Table 2).

**Table 2:** Descriptive statistics for self-efficacy scale.

Item	Mean± SD	Median	Range
How confident do you feel that you can keep the fatigue caused by your disease from interfering with the things you want to do?	5.71 ± 1.7	6	1-10
How confident do you feel that you can keep the physical discomfort or pain of your disease from interfering with the things you want to do?	5.39 ± 1.8	5	1-10
How confident do you feel that you can keep the emotional distress caused by your disease from interfering with the things you want to do?	5.36 ± 1.64	5	1-10
How confident do you feel that you can keep any other symptoms or health problems you have from interfering with the things you want to do?	5.43 ± 1.8	6	1-10
How confident do you feel that you can the different tasks and activities needed to manage your health condition so as to reduce your need to see a doctor?	5.96 ± 1.7	6	2-10
How confident do you feel that you can do things other than just taking medication to reduce how much your illness affects your everyday life?	5.81 ± 1.7	6	1-10

### e-Health Literacy Scale (eHEALS)

The eHEALS results indicated that participants had moderate levels of eHL, with an overall mean score of 3.62 (SD = 0.58, range 1–5). About 59% of participants found the Internet useful for health-related decision-making, while a small proportion felt it was not. Responses indicated that participants had moderate skills in accessing and utilizing health resources online. About 45.6% of participants reported knowing what health resources are available on the internet, and 38.5% knew where to find helpful resources (Table 3).

**Table 3:** Descriptive statistics for e-Health Literacy Scale (eHEALS).

Items	Strongly agree	Agree	Neutral	Disagree	Strongly disagree
I know what health resources are available on the Internet	**19(10.3%)**	**65(35.3%)**	**62(33.7%)**	**31(16.8%)**	**7(3.8%)**
I know where to find helpful health resources on the Internet	**16(8.7%)**	**51(27.7%)**	**72(39.1%)**	**36(19.6%)**	**9(4.9%)**
I know how to find helpful health resources on the Internet	**17(9.2%)**	**54(29.3%)**	**70(38%)**	**34(18.5%)**	**9(4.9%)**
I know how to use the Internet to answer my health questions	**11(6%)**	**71(38.6%)**	**60(32.6%)**	**33(17.9%)**	**9(4.9%)**
I know how to use the health information I find on the Internet to help me	**13(7.1%)**	**62(33.7%)**	**63(34.2%)**	**37(20.1%)**	**9(4.9%)**
I have the skills I need to evaluate the health resources I find on the Internet	**11(6%)**	**53(28.8%)**	**76(41.3%)**	**30(16.3%)**	**14(7.6%)**
I can tell high-quality from low-quality health resources on the Internet	**6(3.3%)**	**66(35.9%)**	**65(35.3%)**	**37(20.1%)**	**10(5.4%)**
I feel confident in using information from the Internet to make health decisions	**13(7.1%)**	**61(33.2%)**	**76(41.3%)**	**26(14.1%)**	**8(4.3%)**

### Quality of Life (QoL)

The overall KDQOL‑36 total score averaged 105 ± 17, with a range of 70–142 and a median of 107 (Q1–Q3: 92–119). The Symptoms/Problems (SP) domain had the highest mean score (39 ± 7), whereas the Burden of Kidney Disease (BKD) domain had the lowest (11 ± 3), indicating a relatively high disease burden. The Effects of Kidney Disease (KDE) and Social Function (SF) domains yielded mean scores of 25 ± 6 and 29 ± 5, respectively. The Physical Component Summary (PCS) and Mental Component Summary (MCS) scores were 12 ± 3 and 21 ± 4, respectively. The wide minimum–maximum ranges observed across all domains indicate substantial inter-individual variability in health-related quality of life among the study population (Table 4).

**Table 4:** QoL subscale scores and averages based on KDQOL-36 questionnaire.

Domain	Mean ± SD	Minimum – Maximum	Median (Q1 – Q3)
**Symptoms/Problems (SP)**	39 ± 7	23–58	39 (34–45)
**Effects of Kidney Disease (KDE)**	25 ± 6	11–35	26 (21–31)
**Burden of Kidney Disease (BKD)**	11 ± 3	4–20	11 (8–14)
**Social Function (SF)**	29 ± 5	16–45	30 (26–32)
**Physical Component Summary (PCS)**	12 ± 3	6–17	12 (10–14)
**Mental Component Summary (MCS)**	21 ± 4	11–34	21 (19–23)
**KDQOL**‑**36 Total Score**	105 ± 17	70–142	107 (92–119)

*Values are presented as* ***Mean ± Standard Deviation (SD)***, ***Minimum–Maximum****, and* ***Median (Q1–Q3)****. KDQOL‑36 = Kidney Disease Quality of Life‑36; SP = Symptoms/Problems; KDE = Effects of Kidney Disease; BKD = Burden of Kidney Disease; SF = Social Function; PCS = Physical Component Summary; MCS = Mental Component Summary; eHEALS = eHealth Literacy Scale; Self‑Efficacy = Patient Self‑Efficacy Scale.*

### Multivariable regression analysis

Multiple linear regression identified several independent predictors of KDQOL-36 total score. The final model was statistically significant (R² = 0.323, adjusted R² = 0.288, p < 0.001), explaining 32.3% of the variance in health-related quality of life (Table 5).

**Table 5:** Multiple linear regression predicting KDQOL-36 total score.

Predictor	B (Unstandardized)	SE	t	p-value	95% CI for B
**Age**	−0.040	0.113	−0.35	0.725	−0.264–0.184
**Marital Status (Married)**	−2.17	2.68	−0.81	0.420	−7.47–3.13
**TOTAL eHEALS**	0.155	0.199	0.78	0.437	−0.238–0.548
**TOTAL Self-Efficacy**	0.141	0.176	0.80	0.424	−0.206–0.487
**Employment (Employed)**	7.14	2.55	2.80	0.006 *	2.10–12.2
**Education (Secondary)**	1.17	2.78	0.42	0.674	−4.32–6.66
**Education (Graduate)**	8.06	2.91	2.77	0.006 *	2.32–13.8
**Diabetes (Yes)**	−5.92	2.52	−2.35	0.020 *	−10.9 – −0.95
**Cardiovascular Disease (Yes)**	−6.81	3.65	−1.87	0.064 †	−14.0–0.39

Multiple R² = 0.323

Adjusted R² = 0.288

Overall model p < 0.001

*Significant at p < 0.05.

†Marginally significant at p < 0.10.

Employment status and higher educational attainment were associated with significantly higher KDQOL-36 scores. Employed participants had, on average, a 7.14-point higher KDQOL-36 score than unemployed participants (B = 7.14, 95% CI: 2.10 to 12.20, p = 0.006). Similarly, participants with graduate-level education had an 8.06-point higher score than those with primary education (B = 8.06, 95% CI: 2.32 to 13.80, p = 0.006).

Conversely, diabetes mellitus was independently associated with poorer quality of life, corresponding to a 5.92-point reduction in the KDQOL-36 score (B = −5.92, 95% CI: −10.90 to −0.95, p = 0.020). Cardiovascular disease showed a borderline negative association (B = −6.81, 95% CI: −14.0 to 0.39, p = 0.064). After adjustment for other covariates, neither age, marital status, e-health literacy, nor self-efficacy remained significant (p > 0.05).

### Correlation with main variables

Spearman’s rho correlation analysis revealed significant associations between age, duration of illness, and various QoL outcomes. Age exhibited a negative correlation with all KDQOL-36 domains (PCS: ρ = 0.002; SP: ρ < 0.001; KDE: ρ = 0.002), indicating that as age increases, QoL scores across all domains decrease. On the other hand, disease duration showed a positive correlation with PCS (ρ = 0.314, p < 0.01), BKD (ρ = 0.405, p < 0.01), Total SP (ρ = 0.442, p < 0.01), and Total KDE (ρ = 0.670, p < 0.01), suggesting that a longer duration of illness is associated with higher scores in these domains.

## Discussion

This study investigated the relationship between eHL, self-efficacy, and QoL among HD patients in Jordan. The primary focus was to explore the socio-demographic factors influencing eHL, examine the impact of self-efficacy on health outcomes, and determine the correlation between eHL, self-efficacy, and QoL. Additionally, the study sought to identify predictors of self-efficacy in this patient group. The findings contribute to a deeper understanding of how these variables interact to influence the overall well-being of HD patients and offer insights into potential interventions.Multivariable regression analysis showed that educational attainment, employment status, and diabetes mellitus were independent predictors of KDQOL-36 total scores. Specifically, participants with graduate-level education and those who were employed reported significantly higher quality-of-life scores, whereas patients with diabetes mellitus reported significantly lower quality-of-life scores. The positive association between higher educational attainment and quality of life may be attributed to greater health knowledge, improved self-management abilities, and an enhanced capacity to access and use health information. Previous evidence shows that individuals with higher levels of education tend to possess stronger digital health competencies and engage more effectively with health-related resources [12]. In addition, broader socioeconomic factors may influence patients’ ability to benefit from available health information and healthcare services, reflecting the ongoing impact of the digital divide on health outcomes [13]. Employment status was also independently associated with better quality of life, which may reflect greater financial stability, social engagement, and psychological well-being. In contrast, diabetes mellitus was independently associated with poorer quality of life, likely due to the increased treatment burden and the higher risk of complications when managing multiple chronic conditions simultaneously. Similar findings have been reported among patients with chronic kidney disease, a greater disease burden associated with poorer health outcomes and reduced quality of life [14].

Interestingly, age, marital status, e-health literacy, and self-efficacy were not independently associated with quality of life in the fully adjusted model. Although e-health literacy and self-efficacy demonstrated significant associations in unadjusted analyses, these associations were attenuated after adjustment for socioeconomic and clinical factors. This suggests that the observed associations between e-health literacy, self-efficacy, and quality of life may be influenced by underlying socioeconomic characteristics, particularly educational attainment and employment status, rather than serving as independent determinants of quality of life.The positive correlation between self-efficacy and QoL in our study aligns with Bandura’s social cognitive theory [5], which argues that self-efficacy, the belief in one’s ability to succeed, is a key determinant of health behavior and outcomes. Patients with higher self-efficacy may be more likely to adhere to prescribed treatments, manage their symptoms effectively, and engage in behaviors that reduce the impact of their disease.Our findings align with other studies in chronic disease populations. For example, a previous study found that self-efficacy was positively associated with QoL in CKD patients [15], leading to fewer hospitalizations and better disease management [16,17].An essential aim of this study was to determine the correlation between eHL, self-efficacy, and QoL. Our analysis revealed strong positive correlations between eHL and self-efficacy, as well as between eHL and QoL. Specifically, patients who were more confident in their ability to manage their disease (higher self-efficacy) and who demonstrated higher levels of eHL reported better physical health (PCS), fewer disease-related symptoms, and lower disease burden (BKD). These findings suggest that improving eHL and self-efficacy can have a synergistic effect on QoL in HD patients.This finding is consistent with Baker et al. (2017) and Zhang et al. (2020), who found that eHL enhances self-management behaviors among patients with chronic disease, leading to better disease outcomes and improved QoL. Moreover, it was found that eHL and self-efficacy contribute to better engagement in health behaviors and more effective management of chronic conditions [18].

### Strengths and limitations

This study has several key strengths. It is among the first to explore the interrelationships among eHL, self-efficacy, and QoL in a Jordanian sample of HD patients, providing valuable insights into psychosocial factors that influence chronic disease management. The use of validated instruments such as the KDQOL-36 ensures the reliability of the findings. Additionally, the study’s focus on socio-demographic factors offers a unique perspective on the contextual determinants of digital health literacy and self-efficacy.However, the study also has several limitations. Its cross-sectional design limits the ability to draw causal conclusions about the relationships among eHL, self-efficacy, and QoL. The study sample was confined to patients from a single healthcare facility, which may not fully represent the diversity of the CKD population in Jordan or other regions. Future studies should incorporate longitudinal designs and larger, more diverse samples to validate these findings. Additionally, other psychosocial factors, such as psychological distress, were not assessed in this study but could provide further insights into the factors influencing eHL and self-efficacy.

### Study implications

The findings of this study have significant implications for healthcare providers, policy makers, and researchers. It underscores the possible advantages of integrating digital literacy programs and self-efficacy training as part of patient care strategies in efforts to improve the health outcomes and overall quality of life of CKD patients undergoing hemodialysis. This could include digital health education programs aimed at improving eHealth skills and self-efficacy training to empower patients to manage their disease more effectively. Targeted interventions for older adults and individuals with lower educational attainment or limited socioeconomic resources may help reduce disparities in e-health literacy and improve equitable access to digital health resources.These findings also suggest that integrating eHL initiatives with self-efficacy training could serve as an effective strategy for improving disease management and overall health outcomes. Additionally, further exploration of the role of socio-demographic factors and digital health literacy in chronic disease management is necessary to develop targeted interventions that address the needs of diverse patient populations.

## Conclusion

This study sought to explore the relationships between eHL, self-efficacy, and QoL among HD patients in Jordan and identify socio-demographic factors influencing these variables. The findings revealed that higher eHL and self-efficacy were significantly associated with better QoL in HD patients. Socio-demographic characteristics such as education, income, and age played crucial roles in shaping both eHL and self-efficacy. The study also found strong correlations between eHL and self-efficacy, which were positively linked with better health outcomes, including fewer disease-related symptoms and reduced disease burden.

The results of this study align with previous research emphasizing the importance of both eHL and self-efficacy in improving QoL among patients with chronic conditions, including CKD. In conclusion, the study underscores the need for healthcare interventions that improve eHL and self-efficacy among HD patients. These factors are crucial for enhancing overall QoL and empowering patients to take a more active role in managing their chronic illness.

## Supporting information

S1 File QOL-ESRD. (XLSX)
