# Supplementary material for: Associations between e-Health literacy, self-efficacy, and quality of life among hemodialysis patients: A cross-sectional study from Northern Jordan
Source: PLoS One. 2026 Aug 4;21(8):e0354170. doi: 10.1371/journal.pone.0354170 (PMC13436721; doi:10.1371/journal.pone.0354170)
Supplement: Supplementary file 1 [file pone.0354170.s003.docx]

**Dear Editor and Reviewers,**

We sincerely thank you for your careful evaluation of our manuscript entitled “Manuscript Title: Associations between e-Health Literacy, Self-Efficacy, and Quality of Life among Hemodialysis Patients: A Cross-Sectional Study from Northern Jordan” and for the constructive comments that helped improve the quality and clarity of the paper. We have carefully revised the manuscript accordingly. All modifications have been clearly marked in the revised manuscript using “Track Changes” function. Below we provide a detailed point-by-point response to each comment.

## Reviewer #1

Comment:

The article lacks novelty. How is it different from other countries? The article would be better if the author adds social conditions in Jordan.

***Response:***

*We appreciate this important comment. To address this concern, we expanded the Introduction to better contextualize the study within the social and socioeconomic conditions of Jordan, particularly regarding disparities in internet access, education, and income that may influence digital health literacy among patients undergoing hemodialysis. The revised manuscript now includes additional discussion describing how socioeconomic conditions and digital access disparities in Jordan may shape patients’ ability to obtain and use online health information. We believe this addition strengthens the contextual relevance and novelty of the study.*

## Reviewer #2

Comment 1: Overly causal interpretation from cross-sectional study.

*Response:*

*We fully agree with the reviewer. The manuscript has been carefully revised to remove causal language throughout the Abstract, Introduction, Discussion, and Conclusion. Terms such as 'effect', 'improve', and 'determine the impact' have been replaced with 'association' or 'relationship' to ensure consistency with the cross-sectional study design.*

Comment 2: Insufficient description of sampling and representativeness.

*Response:*

*We expanded the Methods section to clarify the sampling strategy. Participants were recruited using a consecutive sampling approach in which all eligible hemodialysis patients attending the participating dialysis units during the study period were invited to participate. We also added a discussion of potential selection bias related to the inclusion criterion requiring Arabic literacy and its implications for generalizability.*

Comment 3: Lack of formal sample size justification.

*Response:*

*Thank you for the important comment. We have clarified the sample size justification in the methodology section using the RaoSoft sample size calculator, assuming a 95% confidence level, a 5% margin of error, and a response distribution of 50%. This estimation was informed by national registry data indicating that more than 6,000 patients receive maintenance hemodialysis in Jordan. A total of 184 patients were recruited from four dialysis centers in northern Jordan, representing a substantial proportion of the accessible study population and consistent with sample sizes reported in comparable hemodialysis studies conducted in similar settings. Although the achieved sample size was lower than the calculated requirement, it reflects real-world recruitment constraints within a vulnerable population and supports its adequacy for exploratory and association analyses, while potentially limiting generalizability. This has also been acknowledged in the study limitations. The reference list has been updated accordingly.*

Comment 4: Unclear reporting of multivariable regression.

*Response:*

*We thank the reviewer for this valuable comment. We acknowledge that the reporting of the multivariable regression analysis was unclear in the original manuscript. Although multivariable regression analyses were performed, their results were inadvertently omitted from Table 5 and were therefore not adequately integrated into the Results and Discussion sections. In the revised manuscript, Table 5 has been updated to include multivariable regression analysis used to identify independent predictors of KDQOL-36 total scores. In addition, the Results section has been refined to clearly present the significant predictors identified in the final adjusted model, including educational attainment, employment status, and diabetes mellitus. The Discussion section has also been revised accordingly to align the interpretation of the findings with the multivariable regression results and to avoid overemphasizing associations observed in unadjusted analyses.*

*Furthermore, we clarified the statistical methods section to accurately describe all analyses performed, including the use of non-parametric tests (Spearman correlation, Mann–Whitney U test, and Kruskal–Wallis test) as well as multivariable linear regression to identify independent predictors of health-related quality of life while accounting for potential confounding variables.*

Comment 5: Confusing reporting of statistical metrics and p-values.

Response:

*We carefully reviewed all statistical reporting and corrected instances where p-values were mistakenly labeled as correlation coefficients. Spearman correlation coefficients and p-values are now clearly distinguished throughout the manuscript and tables.*

Comment 6: No consideration of multiple comparisons.

Response:

*We added a statement in the Discussion acknowledging that multiple statistical tests were performed across several quality-of-life domains and demographic variables. We noted that the possibility of type I error due to multiple comparisons should be considered when interpreting the results.*

Comment 7: Unclear definition of 'higher' e-health literacy and self-efficacy.

Response:

*We clarified that the primary analyses treated e-health literacy and self-efficacy as continuous variables rather than dichotomized categories. The wording in the Abstract and Results sections has been revised accordingly.*

Comment 8: Errors and inconsistencies in variable labeling.

Response:

*We appreciate the reviewer for identifying these inconsistencies. The manuscript has been thoroughly proofread and corrected. Specifically, the incorrect reference to 'exercise frequency' has been corrected to 'hemodialysis frequency,' and all tables and descriptive statistics have been checked for consistency.*

Once again, we thank the reviewers for their valuable feedback, which significantly improved the clarity and rigor of the manuscript.

Kind regards

On behalf of all authors

Rawand Khasawneh

Associate Professor of Clinical Pharmacy

Department of Clinical Pharmacy/Faculty of Pharmacy

Jordan University of Science and Technology

Irbid-Jordan

Email: [rakhasawneh@just.edu.jo](mailto:rakhasawneh@just.edu.jo)
