# Supplementary material for: Associations between e-Health literacy, self-efficacy, and quality of life among hemodialysis patients: A cross-sectional study from Northern Jordan
Source: PLoS One. 2026 Aug 4;21(8):e0354170. doi: 10.1371/journal.pone.0354170 (PMC13436721; doi:10.1371/journal.pone.0354170)
Supplement: Peer review history

# Peer review: Decision Letter 0

**DOI:** 10.1371/journal.pone.0354170.r001

**Authors:** Yoshitaka Ishibashi

28 Nov 2025

PONE-D-25-36107

Associations between e-Health Literacy, Self-Efficacy, and Quality of Life among Hemodialysis Patients: A Cross-Sectional Study from Northern Jordan

PLOS ONE

Dear Dr. Khasawneh,

Thank you for submitting your manuscript to PLOS ONE. After careful consideration, we feel that it has merit but does not fully meet PLOS ONE’s publication criteria as it currently stands. Therefore, we invite you to submit a revised version of the manuscript that addresses the points raised during the review process.

Please submit your revised manuscript by Jan 12 2026 11:59PM. If you will need more time than this to complete your revisions, please reply to this message or contact the journal office at plosone@plos.org. When you're ready to submit your revision, log on to https://www.editorialmanager.com/pone/ and select the 'Submissions Needing Revision' folder to locate your manuscript file.

Please include the following items when submitting your revised manuscript:

- A rebuttal letter that responds to each point raised by the academic editor and reviewer(s). You should upload this letter as a separate file labeled 'Response to Reviewers'.
- A marked-up copy of your manuscript that highlights changes made to the original version. You should upload this as a separate file labeled 'Revised Manuscript with Track Changes'.
- An unmarked version of your revised paper without tracked changes. You should upload this as a separate file labeled 'Manuscript'.

If you would like to make changes to your financial disclosure, please include your updated statement in your cover letter. Guidelines for resubmitting your figure files are available below the reviewer comments at the end of this letter.

If applicable, we recommend that you deposit your laboratory protocols in protocols.io to enhance the reproducibility of your results. Protocols.io assigns your protocol its own identifier (DOI) so that it can be cited independently in the future. For instructions see: https://journals.plos.org/plosone/s/submission-guidelines#loc-laboratory-protocols. Additionally, PLOS ONE offers an option for publishing peer-reviewed Lab Protocol articles, which describe protocols hosted on protocols.io. Read more information on sharing protocols at https://plos.org/protocols?utm_medium=editorial-email&utm_source=authorletters&utm_campaign=protocols.

We look forward to receiving your revised manuscript.

Kind regards,

Yoshitaka Ishibashi

Academic Editor

PLOS ONE

Journal Requirements:

When submitting your revision, we need you to address these additional requirements.

1.Please ensure that your manuscript meets PLOS ONE's style requirements, including those for file naming. The PLOS ONE style templates can be found at

https://journals.plos.org/plosone/s/file?id=wjVg/PLOSOne_formatting_sample_main_body.pdf and

https://journals.plos.org/plosone/s/file?id=ba62/PLOSOne_formatting_sample_title_authors_affiliations.pdf

2. In the online submission form you indicate that your data is not available for proprietary reasons and have provided a contact point for accessing this data. Please note that your current contact point is a co-author on this manuscript. According to our Data Policy, the contact point must not be an author on the manuscript and must be an institutional contact, ideally not an individual. Please revise your data statement to a non-author institutional point of contact, such as a data access or ethics committee, and send this to us via return email. Please also include contact information for the third party organization, and please include the full citation of where the data can be found.

3. Please ensure that you refer to Figure 14 in your text as, if accepted, production will need this reference to link the reader to the figure.

4. Please include captions for your Supporting Information files at the end of your manuscript, and update any in-text citations to match accordingly. Please see our Supporting Information guidelines for more information: http://journals.plos.org/plosone/s/supporting-information.

If the reviewer comments include a recommendation to cite specific previously published works, please review and evaluate these publications to determine whether they are relevant and should be cited. There is no requirement to cite these works unless the editor has indicated otherwise.

Additional Editor Comments:

Dear Authors,

Thank you for submitting your manuscript examining the associations of e-health literacy and self-efficacy with quality of life among maintenance hemodialysis patients in northern Jordan. The topic is clinically important and relevant to the dialysis population.

After careful evaluation of the manuscript and consideration of the reviewer’s comments, I believe that your study has potential scientific value. However, both the methodological description and the interpretation of your findings require substantial clarification and revision before the manuscript can be considered for publication.

Please revise the manuscript thoroughly and provide a detailed, point-by-point response to the reviewer comments.

Yoshitaka Ishibashi

[Note: HTML markup is below. Please do not edit.]

Reviewer's Responses to Questions

**Comments to the Author**

1. Is the manuscript technically sound, and do the data support the conclusions?

The manuscript must describe a technically sound piece of scientific research with data that supports the conclusions. Experiments must have been conducted rigorously, with appropriate controls, replication, and sample sizes. The conclusions must be drawn appropriately based on the data presented.

Reviewer #1: Yes

Reviewer #2: No

**********

2. Has the statistical analysis been performed appropriately and rigorously?

Reviewer #1: Yes

Reviewer #2: No

**********

3. Have the authors made all data underlying the findings in their manuscript fully available?

The PLOS Data policy requires authors to make all data underlying the findings described in their manuscript fully available without restriction, with rare exception (please refer to the Data Availability Statement in the manuscript PDF file). The data should be provided as part of the manuscript or its supporting information, or deposited to a public repository. For example, in addition to summary statistics, the data points behind means, medians and variance measures should be available. If there are restrictions on publicly sharing data—e.g. participant privacy or use of data from a third party—those must be specified.

Reviewer #1: Yes

Reviewer #2: No

**********

4. Is the manuscript presented in an intelligible fashion and written in standard English?

PLOS ONE does not copyedit accepted manuscripts, so the language in submitted articles must be clear, correct, and unambiguous. Any typographical or grammatical errors should be corrected at revision, so please note any specific errors here.

Reviewer #1: Yes

Reviewer #2: No

**********

5. Review Comments to the Author

Please use the space provided to explain your answers to the questions above. You may also include additional comments for the author, including concerns about dual publication, research ethics, or publication ethics. (Please upload your review as an attachment if it exceeds 20,000 characters)

Reviewer #1: The purpose of this article is clear, and the method of the analysis is proper, too. Therefore, this article deserves accept. But this article lacks in novelty. How is it different from other countries in the difference of the education and income of Jordan? This article becomes better, if this author adds social conditions in Jordan.

Reviewer #2: Overall assessment

This manuscript reports a cross-sectional study examining the associations of e-health literacy and self-efficacy with quality of life (QoL) among maintenance hemodialysis patients in northern Jordan. The topic is clinically and public-health relevant. However, the current version suffers from several important methodological and reporting issues that substantially weaken the validity of the conclusions.

In particular, the manuscript tends to draw causal inferences (e.g., suggesting that interventions on e-health literacy and self-efficacy will improve QoL) that are not supported by the cross-sectional design. Moreover, the key claims regarding the impact of e-health literacy and self-efficacy on QoL are not backed by adequately reported multivariable analyses that adjust for major confounders.

Overall, I believe that a major revision is required before the manuscript can be considered for publication.

Major comments

1. Overly causal interpretation of findings from a cross-sectional study

The study is clearly cross-sectional, based on measurements at a single time point. Nevertheless, the Abstract and main text repeatedly use causal language, such as “interventions targeting e-health literacy and self-efficacy can significantly improve QoL” and “improving e-health literacy… might enhance self-management.”

o The conclusions should be reframed in terms of association rather than effect or improvement.

o Phrases like “determine the effect” should be replaced with “assess the association” throughout the Introduction and Discussion.

2. Insufficient description of sampling and representativeness

The sample is described as 184 hemodialysis patients from four centers in northern Jordan, but:

o The sampling strategy (consecutive sampling, convenience sampling, or complete enumeration) is not clearly documented.

o The participation rate (number consented / number eligible) is not reported, so the extent of selection bias cannot be evaluated.

o The inclusion criterion of being able to read and write Arabic may systematically exclude older or less literate patients, yet this potential selection bias and its impact on generalizability are not discussed.

The authors should more fully describe the sampling process and critically discuss the representativeness and external validity of their findings.

3. Lack of formal sample size justification and power considerations

The authors refer to Green (1991) to argue that the sample size is adequate. However:

o No a priori sample size calculation based on expected effect size, significance level, and power is presented.

o Given the number of predictors and multiple QoL domains, simple rules-of-thumb are not sufficient.

At least for the main hypotheses (e.g., associations between e-health literacy and QoL), the authors should present a more formal justification of the sample size and the detectable effect size.

4. Unclear reporting of multivariable regression and lack of adjustment for confounders

The Methods section states that linear regression analyses were performed. However:

o The Results section is dominated by Spearman correlations and Mann–Whitney/Kruskal–Wallis tests, with almost no explicit reporting of the regression models.

o It remains unclear which variables were included in each model, what the outcome variables were, and what the regression coefficients, 95% confidence intervals, and p-values were.

o Age, education, income, and comorbidities (e.g., diabetes, CVD) are clearly associated with QoL, as the authors themselves show, yet it is not shown whether the associations of e-health literacy and self-efficacy with QoL remain after adjustment for these factors.

Given that the main conclusions rely on these associations, the multivariable models must be fully and transparently reported (model specification, variable selection criteria, assessment of multicollinearity, regression estimates with 95%CIs, etc.).

5. Confusing reporting of statistical metrics and p-values

In several places, the manuscript reports statements such as “Age exhibited a negative correlation… (PCS: ρ = 0.002; SP: ρ < 0.001…)”.

o By convention, ρ denotes the Spearman correlation coefficient, which should range between -1 and 1.

o The reported numbers (0.002, <0.001) are clearly p-values, not correlation coefficients.

Similarly, in Table 6, the columns “Mean Rank” and “ρ-value” are presented in a way that makes it difficult for readers to distinguish between effect sizes and p-values.

The authors should carefully revise the terminology and tables to clearly distinguish correlation coefficients (ρ) from p-values, and to label all statistics correctly and consistently.

6. No consideration of multiple comparisons

The manuscript performs numerous statistical tests across five QoL domains and many socio-demographic and clinical variables and reports many p-values.

o No adjustment for multiple testing (e.g., Bonferroni, Holm, or FDR) appears to have been applied.

o As a result, some of the “significant” findings may simply be due to chance.

At minimum, the authors should distinguish between primary and exploratory analyses and explicitly discuss the issue of multiple comparisons as a limitation.

o

7. Unclear definition of “higher” e-health literacy and self-efficacy

In the Abstract, the authors describe “patients with higher e-health literacy (75%)” and “higher self-efficacy (80%)”, implying a dichotomization. However:

o The main text does not clearly specify how “higher” was defined (e.g., based on median split, established cut-off, or other rationale).

o Arbitrary dichotomization of continuous scales reduces statistical power and may introduce bias.

If such categorizations are used, the cut-off values and their justification must be explicitly stated. Preferably, the primary analyses should treat these scales as continuous variables.

8. Errors and inconsistencies in variable labeling and descriptive statistics

For example, in Table 1, the text states that “4.3% exercised twice a week, 95.1% exercised three times a week,” which appears to be a mislabeling of hemodialysis frequency rather than physical exercise. Moreover, in the main text it is written that “4.3% (n=8) exercised twice a week, 95.1% (n=175) exercised three times a 156 week, and 0.5% (n=1) exercised four times a week,” whereas in Table 1 the same percentages are presented under the category of “dialysis,” clearly indicating that these figures actually refer to dialysis sessions, not exercise.

Such errors and inconsistencies raise concerns about data management and the reliability of the reported results. A thorough proofreading and consistency check of all tables and text is necessary.

Minor comments

1. Terminology should be standardized throughout the manuscript (e.g., “hemodialysis” vs HD, the use of “HD” abbreviation, etc.).

2. More detailed information on the psychometric properties of the Arabic versions of the e-health literacy, self-efficacy, and QoL scales would strengthen the Methods section (e.g., Cronbach’s alpha, previous validation studies).

3. For key Likert-type items, presenting the distribution (e.g., percentages in each category) in addition to means and SDs would improve interpretability.

4. There are several grammatical and typographical errors in the English text; professional language editing is recommended.

**********

6. PLOS authors have the option to publish the peer review history of their article (what does this mean?). If published, this will include your full peer review and any attached files.

If you choose “no”, your identity will remain anonymous but your review may still be made public.

**Do you want your identity to be public for this peer review?**  For information about this choice, including consent withdrawal, please see our Privacy Policy.

Reviewer #1: No

Reviewer #2: No

**********

[NOTE: If reviewer comments were submitted as an attachment file, they will be attached to this email and accessible via the submission site. Please log into your account, locate the manuscript record, and check for the action link "View Attachments". If this link does not appear, there are no attachment files.]

To ensure your figures meet our technical requirements, please review our figure guidelines: https://journals.plos.org/plosone/s/figures

You may also use PLOS’s free figure tool, NAAS, to help you prepare publication quality figures: https://journals.plos.org/plosone/s/figures#loc-tools-for-figure-preparation.

NAAS will assess whether your figures meet our technical requirements by comparing each figure against our figure specifications.

# Author comment: Author response to Decision Letter 1

**DOI:** 10.1371/journal.pone.0354170.r002

18 Jun 2026

Dear Editor and Reviewers,

We sincerely thank you for your careful evaluation of our manuscript entitled “Manuscript Title: Associations between e-Health Literacy, Self-Efficacy, and Quality of Life among Hemodialysis Patients: A Cross-Sectional Study from Northern Jordan” and for the constructive comments that helped improve the quality and clarity of the paper. We have carefully revised the manuscript accordingly. All modifications have been clearly marked in the revised manuscript using “Track Changes” function. Below we provide a detailed point-by-point response to each comment.

Reviewer #1

Comment:

The article lacks novelty. How is it different from other countries? The article would be better if the author adds social conditions in Jordan.

Response:

We appreciate this important comment. To address this concern, we expanded the Introduction to better contextualize the study within the social and socioeconomic conditions of Jordan, particularly regarding disparities in internet access, education, and income that may influence digital health literacy among patients undergoing hemodialysis. The revised manuscript now includes additional discussion describing how socioeconomic conditions and digital access disparities in Jordan may shape patients’ ability to obtain and use online health information. We believe this addition strengthens the contextual relevance and novelty of the study.

Reviewer #2

Comment 1: Overly causal interpretation from cross-sectional study.

Response:

We fully agree with the reviewer. The manuscript has been carefully revised to remove causal language throughout the Abstract, Introduction, Discussion, and Conclusion. Terms such as 'effect', 'improve', and 'determine the impact' have been replaced with 'association' or 'relationship' to ensure consistency with the cross-sectional study design.

Comment 2: Insufficient description of sampling and representativeness.

Response:

We expanded the Methods section to clarify the sampling strategy. Participants were recruited using a consecutive sampling approach in which all eligible hemodialysis patients attending the participating dialysis units during the study period were invited to participate. We also added a discussion of potential selection bias related to the inclusion criterion requiring Arabic literacy and its implications for generalizability.

Comment 3: Lack of formal sample size justification.

Response:

Thank you for the important comment. We have clarified the sample size justification in the methodology section using the RaoSoft sample size calculator, assuming a 95% confidence level, a 5% margin of error, and a response distribution of 50%. This estimation was informed by national registry data indicating that more than 6,000 patients receive maintenance hemodialysis in Jordan. A total of 184 patients were recruited from four dialysis centers in northern Jordan, representing a substantial proportion of the accessible study population and consistent with sample sizes reported in comparable hemodialysis studies conducted in similar settings. Although the achieved sample size was lower than the calculated requirement, it reflects real-world recruitment constraints within a vulnerable population and supports its adequacy for exploratory and association analyses, while potentially limiting generalizability. This has also been acknowledged in the study limitations. The reference list has been updated accordingly.

Comment 4: Unclear reporting of multivariable regression.

Response:

We thank the reviewer for this valuable comment. We acknowledge that the reporting of the multivariable regression analysis was unclear in the original manuscript. Although multivariable regression analyses were performed, their results were inadvertently omitted from Table 5 and were therefore not adequately integrated into the Results and Discussion sections. In the revised manuscript, Table 5 has been updated to include multivariable regression analysis used to identify independent predictors of KDQOL-36 total scores. In addition, the Results section has been refined to clearly present the significant predictors identified in the final adjusted model, including educational attainment, employment status, and diabetes mellitus. The Discussion section has also been revised accordingly to align the interpretation of the findings with the multivariable regression results and to avoid overemphasizing associations observed in unadjusted analyses.

Furthermore, we clarified the statistical methods section to accurately describe all analyses performed, including the use of non-parametric tests (Spearman correlation, Mann–Whitney U test, and Kruskal–Wallis test) as well as multivariable linear regression to identify independent predictors of health-related quality of life while accounting for potential confounding variables.

Comment 5: Confusing reporting of statistical metrics and p-values.

Response:

We carefully reviewed all statistical reporting and corrected instances where p-values were mistakenly labeled as correlation coefficients. Spearman correlation coefficients and p-values are now clearly distinguished throughout the manuscript and tables.

Comment 6: No consideration of multiple comparisons.

Response:

We added a statement in the Discussion acknowledging that multiple statistical tests were performed across several quality-of-life domains and demographic variables. We noted that the possibility of type I error due to multiple comparisons should be considered when interpreting the results.

Comment 7: Unclear definition of 'higher' e-health literacy and self-efficacy.

Response:

We clarified that the primary analyses treated e-health literacy and self-efficacy as continuous variables rather than dichotomized categories. The wording in the Abstract and Results sections has been revised accordingly.

Comment 8: Errors and inconsistencies in variable labeling.

Response:

We appreciate the reviewer for identifying these inconsistencies. The manuscript has been thoroughly proofread and corrected. Specifically, the incorrect reference to 'exercise frequency' has been corrected to 'hemodialysis frequency,' and all tables and descriptive statistics have been checked for consistency.

Once again, we thank the reviewers for their valuable feedback, which significantly improved the clarity and rigor of the manuscript.

Kind regards

On behalf of all authors

Rawand Khasawneh

Associate Professor of Clinical Pharmacy

Department of Clinical Pharmacy/Faculty of Pharmacy

Jordan University of Science and Technology

Irbid-Jordan

Email: rakhasawneh@just.edu.jo

Attachment Submitted filename: ESRD_PLOs one_Resposne to reviewers _FINAL.docx

# Editor report: Decision Letter 1

**DOI:** 10.1371/journal.pone.0354170.r003

**Authors:** Yoshitaka Ishibashi

5 Jul 2026

Associations between e-Health Literacy, Self-Efficacy, and Quality of Life among Hemodialysis Patients: A Cross-Sectional Study from Northern Jordan

PONE-D-25-36107R1

Dear Dr. Khasawneh,

We’re pleased to inform you that your manuscript has been judged scientifically suitable for publication and will be formally accepted for publication once it meets all outstanding technical requirements.

Within one week, you’ll receive an e-mail detailing the required amendments. When these have been addressed, you’ll receive a formal acceptance letter and your manuscript will be scheduled for publication.

An invoice will be generated when your article is formally accepted. Please note, if your institution has a publishing partnership with PLOS and your article meets the relevant criteria, all or part of your publication costs will be covered. Please make sure your user information is up-to-date by logging into Editorial Manager at Editorial Manager® and clicking the ‘Update My Information' link at the top of the page. For questions related to billing, please contact billing support.

If your institution or institutions have a press office, please notify them about your upcoming paper to help maximize its impact. If they’ll be preparing press materials, please inform our press team as soon as possible -- no later than 48 hours after receiving the formal acceptance. Your manuscript will remain under strict press embargo until 2 pm Eastern Time on the date of publication. For more information, please contact onepress@plos.org.

Kind regards,

Yoshitaka Ishibashi

Academic Editor

PLOS One

# Editor report: Acceptance letter

**DOI:** 10.1371/journal.pone.0354170.r004

**Authors:** Yoshitaka Ishibashi

PONE-D-25-36107R1

PLOS One

Dear Dr. Khasawneh,

I'm pleased to inform you that your manuscript has been deemed suitable for publication in PLOS One. Congratulations! Your manuscript is now being handed over to our production team.

At this stage, our production department will prepare your paper for publication. This includes ensuring the following:

* All references, tables, and figures are properly cited

* All relevant supporting information is included in the manuscript submission,

* There are no issues that prevent the paper from being properly typeset

You will receive further instructions from the production team, including instructions on how to review your proof when it is ready. Please keep in mind that we are working through a large volume of accepted articles, so please give us a few days to review your paper and let you know the next and final steps.

Lastly, if your institution or institutions have a press office, please let them know about your upcoming paper now to help maximize its impact. If they'll be preparing press materials, please inform our press team within the next 48 hours. Your manuscript will remain under strict press embargo until 2 pm Eastern Time on the date of publication. For more information, please contact onepress@plos.org.

You will receive an invoice from PLOS for your publication fee after your manuscript has reached the completed accept phase. If you receive an email requesting payment before acceptance or for any other service, this may be a phishing scheme. Learn how to identify phishing emails and protect your accounts at https://explore.plos.org/phishing.

If we can help with anything else, please email us at customercare@plos.org.

Thank you for submitting your work to PLOS ONE and supporting open access.

Kind regards,

PLOS ONE Editorial Office Staff

on behalf of

Dr. Yoshitaka Ishibashi

Academic Editor

PLOS One
